# Different Sets of Post-Embryonic Development Genes Are Conserved or Lost in Two Caryophyllales Species (*Reaumuria soongorica* and *Agriophyllum squarrosum*)

**DOI:** 10.1371/journal.pone.0148034

**Published:** 2016-01-27

**Authors:** Pengshan Zhao, Jiwei Zhang, Xin Zhao, Guoxiong Chen, Xiao-Fei Ma

**Affiliations:** 1 Key Laboratory of Stress Physiology and Ecology in Cold and Arid Regions, Gansu Province, Cold and Arid Regions Environmental and Engineering Research Institute, Chinese Academy of Sciences, Lanzhou, P.R. China; 2 Shapotou Desert Research & Experiment Station, Cold and Arid Regions Environmental and Engineering Research Institute, Chinese Academy of Sciences, Lanzhou, P.R. China; INRA, FRANCE

## Abstract

*Reaumuria soongorica* and sand rice (*Agriophyllum squarrosum*) belong to the clade of Caryophyllales and are widely distributed in the desert regions of north China. Both plants have evolved many specific traits and adaptation strategies to cope with recurring environmental threats. However, the genetic basis that underpins their unique traits and adaptation remains unknown. In this study, the transcriptome data of *R*. *soongorica* and sand rice were compared with three other species with previously sequenced genomes (*Arabidopsis thaliana*, *Oryza sativa*, and *Beta vulgaris*). Four different gene sets were identified, namely, the genes conserved in both species, those lost in both species, those conserved in *R*. *soongorica* only, and those conserved in sand rice only. Gene ontology showed that post-embryonic development genes (PEDGs) were enriched in all gene sets, and different sets of PEDGs were conserved or lost in both the *R*. *soongorica* and sand rice genomes. Expression profiles of *Arabidopsis* orthologs further provided some clues to the function of the species-specific conserved PEDGs. Such orthologs included *LEAFY PETIOLE*, which could be a candidate gene involved in the development of branch priority in sand rice.

## Introduction

*Reaumuria soongorica* and sand rice (*Agriophyllum squarrosum*) belong to the clade of Caryophyllales and are widely distributed in the arid regions of north China [1–3]. *R*. *soongorica* (2*n* = 22; genome size = 778 Mb) is a perennial xeric shrub and a constructive, dominant species populating the zonal landscape in desert ecosystems [1,4,5]. During adaptation to recurring environmental threats and continuing desertification, *R*. *soongorica* has evolved many specific traits and physiological changes, such as its extremely thick cuticle, hollow stomata, the presence of a salt gland, and accumulation of some low-molecular-weight metabolites [1,4,6]. Another remarkable characteristic of *R*. *soongorica* is resurrection. *R*. *soongorica* can enter temporal dormancy when water is limited but resumes its original active functions when water becomes available [6].

**Fig 1:**
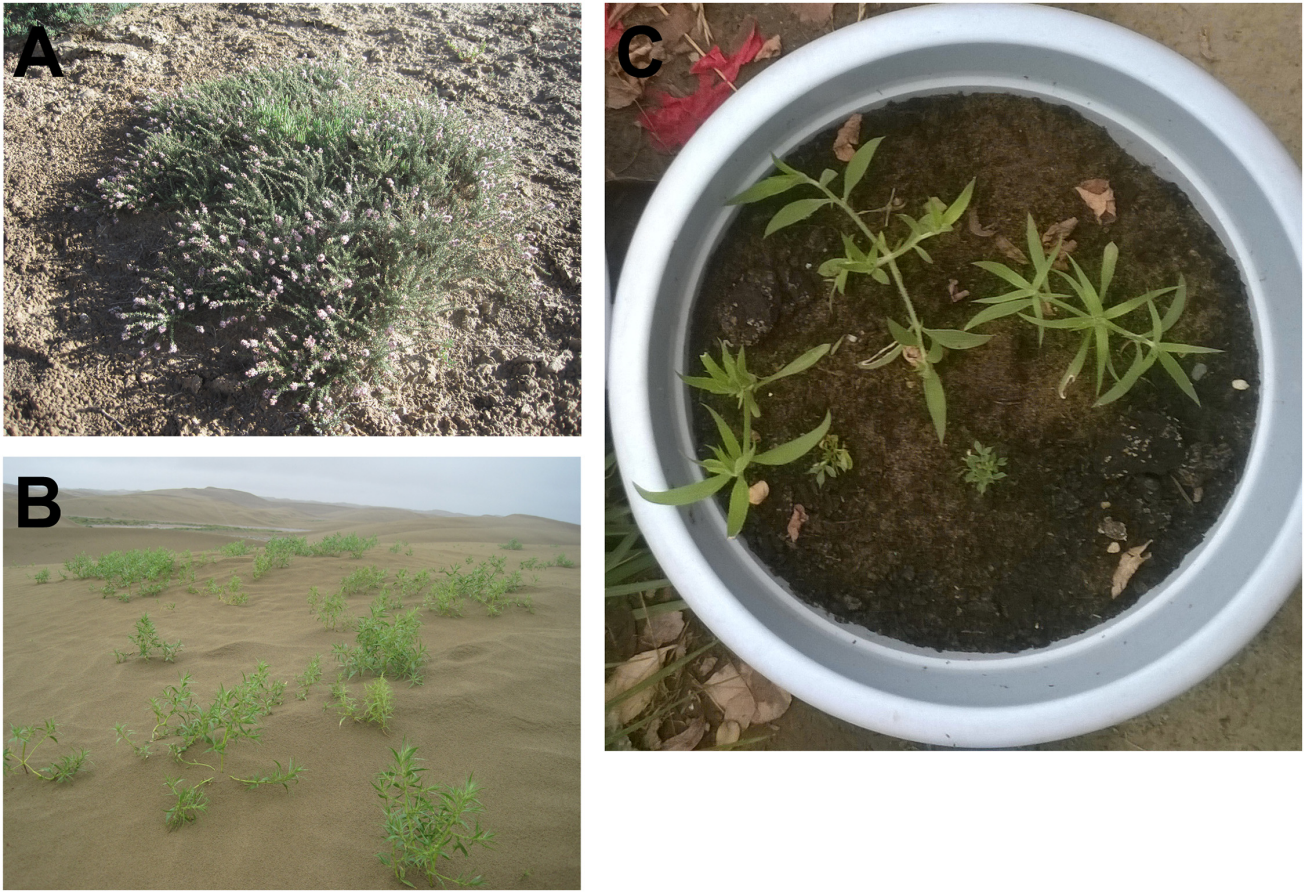
Habitat and shoot architecture. Habitat (A and B) of *R*. *soongorica* and sand rice in China; (C) the branch priority of sand rice.

Sand rice (2*n* = 18; genome size = 714–758 Mb) is branched later than *R*. *soongorica* within Caryophyllales and is a pioneer annual psammophyte endemic to the mobile and semi-mobile sand dunes, which differ from the habitat of *R*. *soongorica* (Fig 1A and 1B; [3,7]). Sand rice has evolved many strategies to adapt to sand dune surface environment, including rapid root growth after germination, long hypocotyl, and pronounced drought and heat tolerance [3,7]. In the seedling stage, the shoot growth of sand rice assumes a striking pattern [7]. Two lateral branches emerge first, while the main stem is inhibited; the main stem then develops later with the second pair of branches (Fig 1C). This special shoot architecture, called branch priority, is crucial to survival against violent wind damage [7].

*R*. *soongorica* and sand rice possess unique phenotypic features and ecological adaptations, rendering them as models to investigate the evolution of stress response strategies and the genetic basis of important traits essential to survival in the harsh environment of the desert ecosystem. The transcriptomes of *R*. *soongorica* and sand rice have been assembled using Illumina sequencing [1,3]. Sets of candidate genes related to abiotic stress events or tolerance have been identified previously through comparisons with the expression profiles of the orthologs in *Arabidopsis thaliana*, as well as digital gene expression profiling [3,4]. However, many of the fundamental questions, including those on speciation and the genetic basis for unique traits, remain open for study. Genome structure and evolution are crucial for plant phenotypic diversity and speciation [8,9]. Characterization of the orthologous gene composition among different species is a powerful strategy in analyzing gene conservation and loss during speciation and adaption to the habitat [10–14]. In the present study, gene conservation and loss in *R*. *soongorica* and sand rice were unraveled by comparing their proteins with those of three other sequenced plants: *A*. *thaliana*, *Oryza sativa* (rice), and *Beta vulgaris* (sugar beet). Gene ontology (GO) showed that post-embryonic development genes (PEDGs) were enriched in all four gene sets. The expression profiles of the *Arabidopsis* orthologs were further acquired to gain more insight into the function of the species-specific PEDGs.

## Materials and Methods

### Orthologous gene clusters

The protein sequences of *A*. *thaliana* and rice were downloaded from the TAIR10 release (www.arabidopsis.org) and Rice Genome Annotation Project (http://rice.plantbiology.msu.edu/). Information on sugar beet proteins was obtained from Dohm et al. [15] (http://bvseq.molgen.mpg.de/Genome/Download/RefBeet-1.1/). For *R*. *soongorica* and sand rice, unigenes from our two previous publications [1,3] were downloaded and the ORFs were analyzed using the Getorf program from both sides to obtain the protein sequence (http://emboss.sourceforge.net/apps/cvs/emboss/apps/getorf.html). Orthologous gene clusters were constructed according to the method of Golicz et al. [11]. In brief, the genes conserved among the five species (*A*. *thaliana*, *O*. *sativa*, *B*. *vulgaris*, *R*. *soongorica*, and *A*. *squarrosum*) were screened by obtaining the reciprocal best hit (RBH, Blastp program with an e-value < 1e-5) between *Arabidopsis* genes and each gene of the four other species. Orthologous genes were assigned into four types of Clusters, particularly, from 2 to 5, and each sub-cluster contained at least an *Arabidopsis* gene and one gene from the remaining species.

### Analysis of gene conservation and loss in R. soongorica and sand rice

*B*. *vulgaris* is phylogenetically close to *R*. *soongorica* and sand rice within the Caryophyllales family [3]. To be strict, only the sub-clusters comprising at least three genes (one *Arabidopsis* gene, one rice ortholog, and one sugar beet gene) were used to identify the conserved and lost genes in *R*. *soongorica* and sand rice. In particular, 1,113 and 1,666 sub-clusters included the rice and sugar beet genes, respectively, in Cluster3. Only 560 overlapping sub-clusters included both rice and sugar beet genes.

### GO annotation

The representative *Arabidopsis* gene in each identified sub-cluster was subjected to GO annotation using AgriGO with default setting [16]. The GO type was Plant GO slim. GO enrichment was analyzed using the hypergeometric test and corrected by Yekutieli (FDR under dependency). The enrichment thresholds of FDR were less than 0.01 in gene sets conserved and lost in *R*. *soongorica* and sand rice and those conserved only in *R*. *soongorica*. For sand rice-specific conserved gene set, the threshold of FDR was less than 0.05. The pairwise comparisons in different gene sets were done using the Student’s *t*-test.

### Expression profile analysis

The Affymetrix GeneChip array data of global gene expression profiles during *Arabidopsis* seed development were downloaded from the NCBI GEO database (GSE680; [17]). The raw data were standardized by RMA with Genespring software (version 12.5, Agilent). Heat maps for the different sets of PEDGs were generated using Multi-Experiment viewer software (MeV, version 4.8.1) with unsupervised hierarchical clustering analysis.

## Results

### Identification of the genes conserved or lost in R. soongorica and sand rice

**Fig 2:**
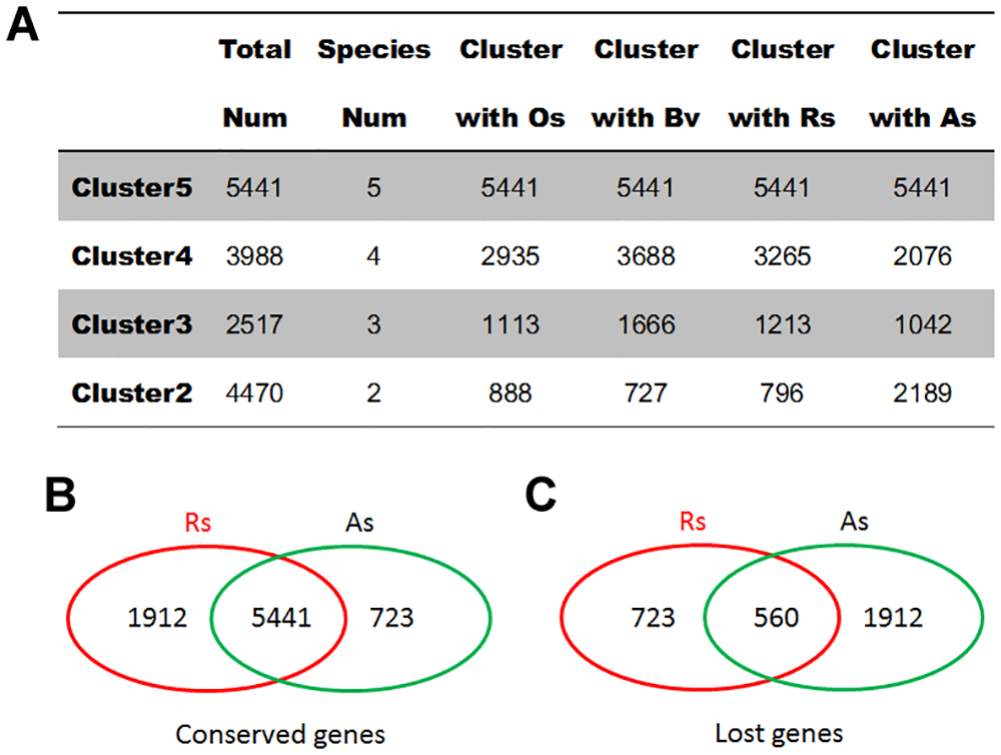
Genes conserved and lost in *R*. *soongorica* and sand rice. (A) Four kinds of Clusters with different numbers of orthologs are showed. In particular, 4470 sub-clusters were assigned into Cluster2, of which 888 sub-clusters contained the *A*. *thaliana* and *O*. *sativa* genes; (B-C) A total of 5,441 genes were conserved and 560 genes were lost in both *R*. *soongorica* and sand rice. Meanwhile, 1912 genes were conserved in *R*. *soongorica* but lost in sand rice, whereas 723 genes were conserved in sand rice but lost in *R*. *soongorica*. Os, *O*. *sativa*; Bv, *B*. *vulgaris*; Rs, *R*. *soongorica*; As, *A*. *squarrosum*.

Four types of Clusters (2–5) were constructed, and a total of 16,416 sub-clusters were identified (Fig 2). In Cluster2, each sub-cluster contained only two genes: one from *Arabidopsis* and another from the remaining four species. To be strict and logical, Cluster2 was not included in the screening of conserved and lost genes. Sugar beet is a representative species of the Caryophyllales family, and it has emerged after *R*. *soongorica* but before sand rice speciation [3,15]. The recently assembled genome of sugar beet could shed more light on the genome evolution of *R*. *soongorica* and sand rice. The sub-clusters comprising at least three genes from *Arabidopsis*, rice, and sugar beet were then analyzed. In Cluster5, all sub-clusters (5,441) included five genes, representing one ortholog from each five species. In Cluster3, 1,113 and 1,666 sub-clusters contained one rice ortholog and one sugar beet gene, respectively. The number of shared sub-clusters containing both rice and sugar beet genes was 560. Therefore, 5,441 genes were conserved and 560 genes were lost in both *R*. *soongorica* and sand rice, as compared with the three remaining species (*A*. *thaliana*, *O*. *sativa*, and *B*. *vulgaris*).

In Cluster4, each sub-cluster contained four genes from four different species (Fig 2). Among these sub-clusters, 2,935 and 3,688 included the rice and sugar beet orthologous genes, respectively. A total of 2,635 shared sub-clusters were found to contain only one ortholog from *R*. *soongorica* or sand rice. Detailed analysis identified the sets of species-specific conserved and lost genes. A total of 1912 genes were conserved in *R*. *soongorica* but lost in sand rice, whereas 723 genes were conserved in sand rice but lost in *R*. *soongorica*.

### Characteristics of the different sets of conserved and lost genes

**Fig 3:**
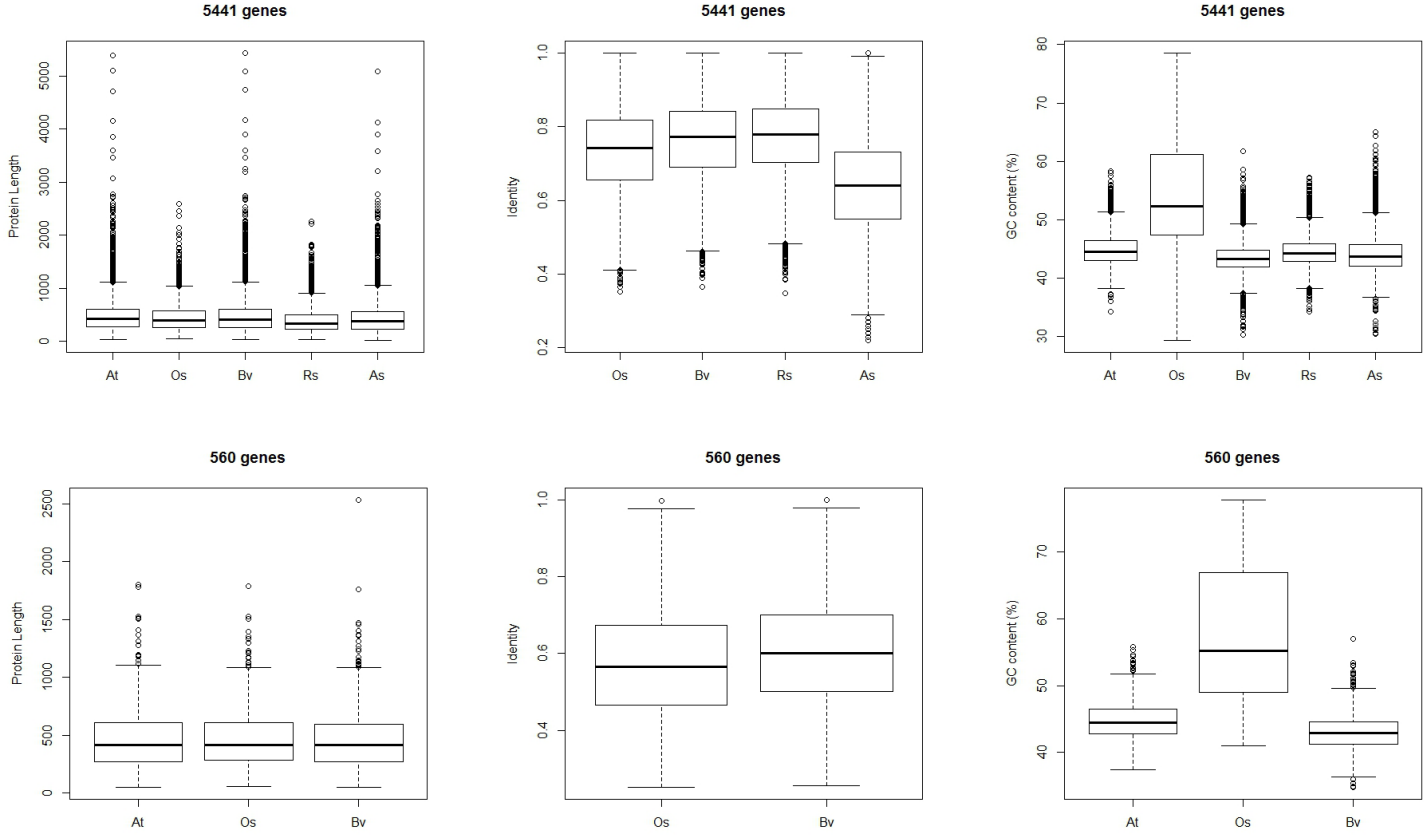
Characteristics of the conserved (5441 genes) and lost (560 genes) gene sets common to both *R*. *soongorica* and sand rice. At, *A*. *thaliana*; Os, *O*. *sativa*; Bv, *B*. *vulgaris*; Rs, *R*. *soongorica*; As, *A*. *squarrosum*.

**Fig 4:**
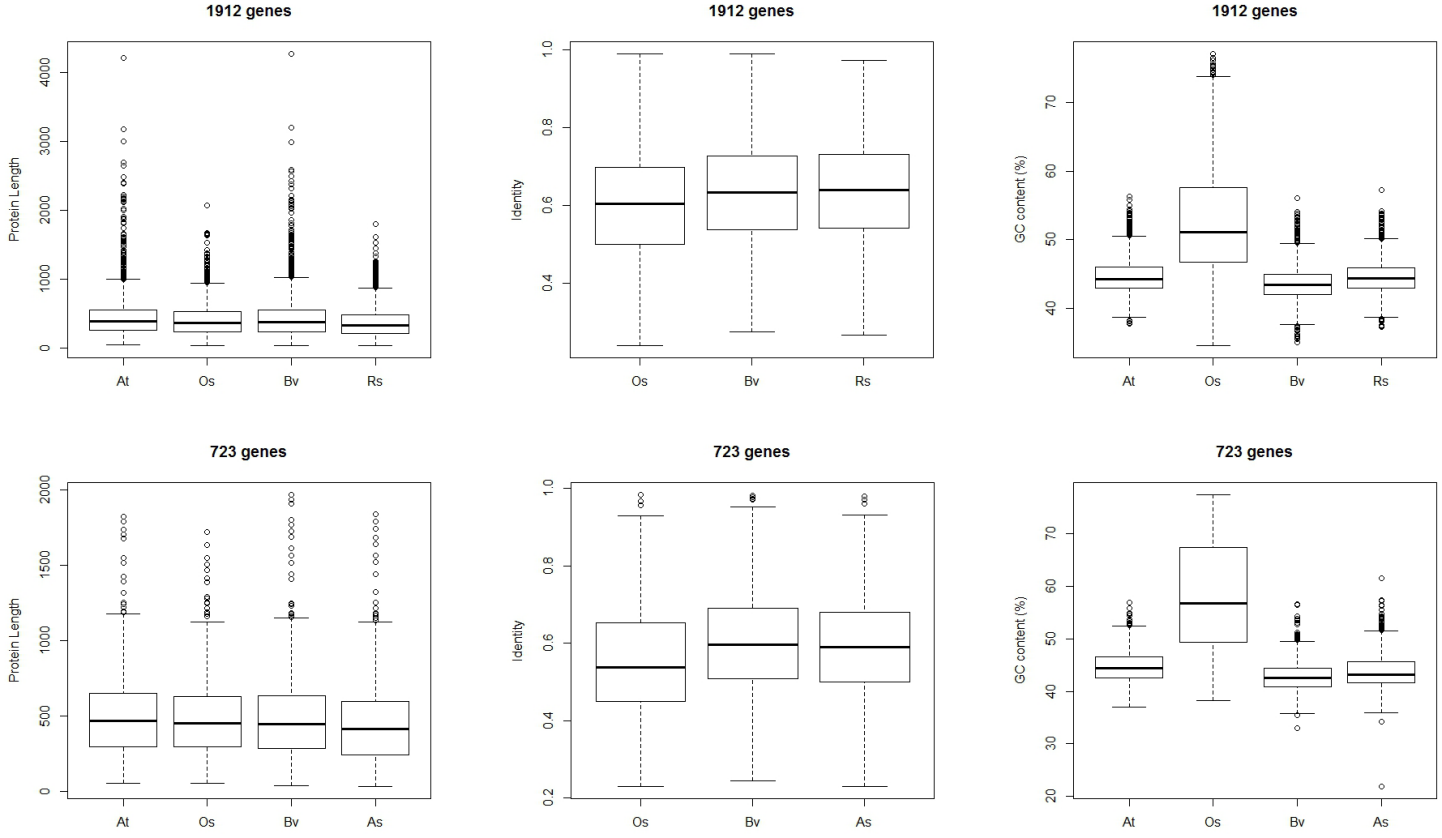
Characteristics of the species-specific conserved and lost gene sets. A total of 1912 genes were conserved in *R*. *soongorica* but lost in sand rice, whereas 723 genes were conserved in sand rice but lost in *R*. *soongorica*.

The protein length, protein identities with the *Arabidopsis* orthologs, and transcript GC contents of the genes in each conserved or lost sub-cluster were characterized in detail (Figs 3 and 4). For the 5,441 genes conserved in both *R*. *soongorica* and sand rice, the protein lengths in *R*. *soongorica* and sand rice were significantly shorter than those in *Arabidopsis* and sugar beet (Fig 3). Unexpectedly, the average protein sequence identity with the *Arabidopsis* orthologous protein was 63.45% in sand rice, which was even lower than that in rice. The mean GC content of the rice transcripts (54.61%) was the highest among the five species. Although the GC ratios ranged from 43.46% to 44.88%, pairwise tests showed marked differences among the remaining four species (*p* < 0.01, S1 Table). For the 560 genes lost in both *R*. *soongorica* and sand rice, similar patterns were found in the protein identities and GC contents, although only the proteins from three species were included in each sub-cluster of this set (Fig 3). The protein lengths of the 560 genes from *Arabidopsis*, rice, and sugar beet were relatively similar (*p* > 0.05), which differed from the situation in the gene set conserved in both *R*. *soongorica* and sand rice.

In the species-specific conserved or lost gene sets (Fig 4), the protein lengths in *R*. *soongorica* (*p* < 0.01) and sand rice (*p* < 0.05) were shorter compared with those in the other three species. The protein identities of *R*. *soongorica* and sand rice with *Arabidopsis* proteins showed no difference from that of sugar beet in the 1912 (*p* = 0.23) and 723 gene sets (*p* = 0.41), respectively, which were consistent with their phylogenetic relationship [3]. The GC contents resembled the results of 5441 and 560 gene sets, except for the comparison between the *R*. *soongorica* and *Arabidopsis* genes in the 1912 gene set (*p* = 0.26).

### GO annotations of the different sets of conserved and lost genes

**Fig 5:**
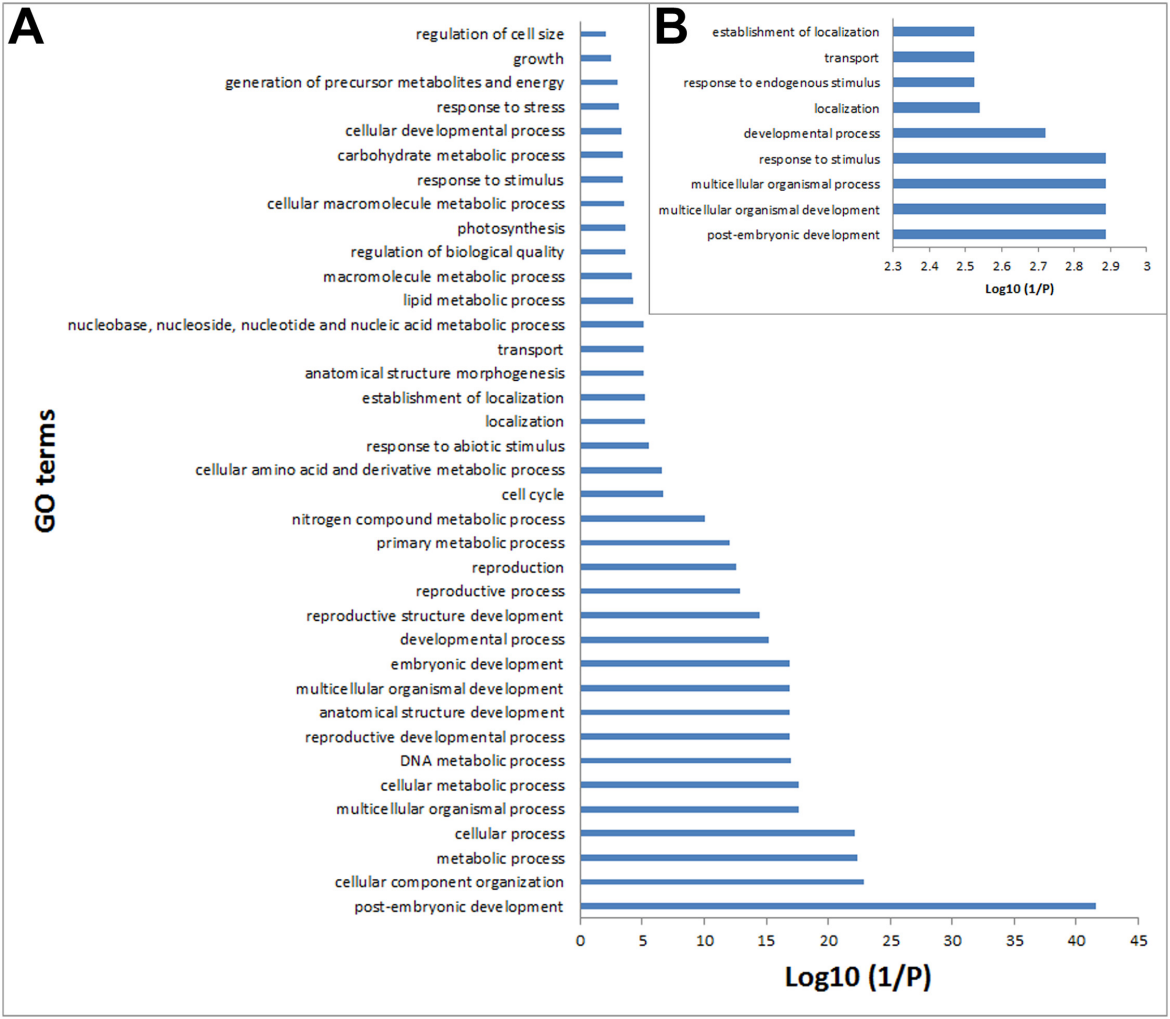
GO biological process term annotation of the conserved and lost genes common to both *R*. *soongorica* and sand rice. The (A) 5441 conserved and (B) 560 lost genes common to both species.

**Fig 6:**
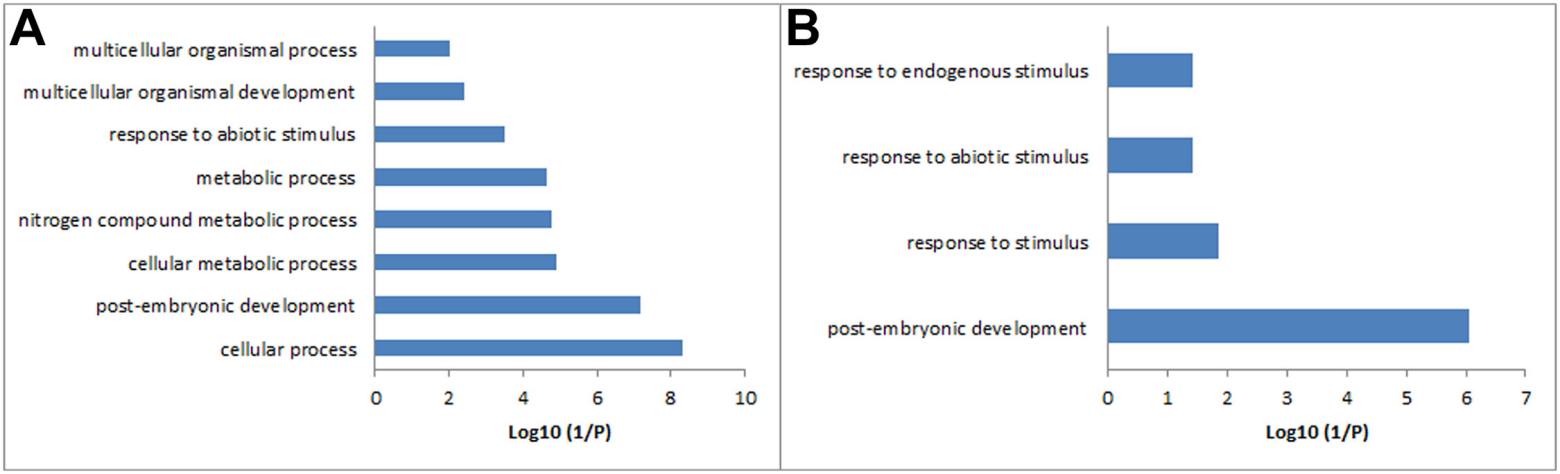
GO biological process annotation of the species-specific conserved genes. (A) Annotation of the 1912 *R*. *soongorica* conserved genes; (B) annotation of the 723 sand rice conserved genes.

To gain more insight into the functions of the genes clustered in the different sets, the representative *Arabidopsis* ortholog in each sub-cluster was subjected to GO term enrichment using the AgriGO program [16]. A total of 91 GO terms were strongly enriched among all gene sets. In the gene set conserved in both *R*. *soongorica* and sand rice, 37 terms were overrepresented in biological process, 12 in molecular functions, and 36 in cellular component categories (*p* < 0.01). The GO categories “post-embryonic development”, “catalytic activity”, and “intracellular” were the most enriched (Fig 5A and S1 Fig). By narrowing the GO enrichment terms into the biological process category, “post-embryonic development” was identified to be shared among common and species-specific conserved and lost gene sets (Figs 5 and 6). These results demonstrated that the different sets of genes involved in post-embryonic development were conserved during the speciation and adaptation of *R*. *soongorica* and sand rice.

### Expression profiles of Arabidopsis post-embryonic development orthologs during seed development

**Fig 7:**
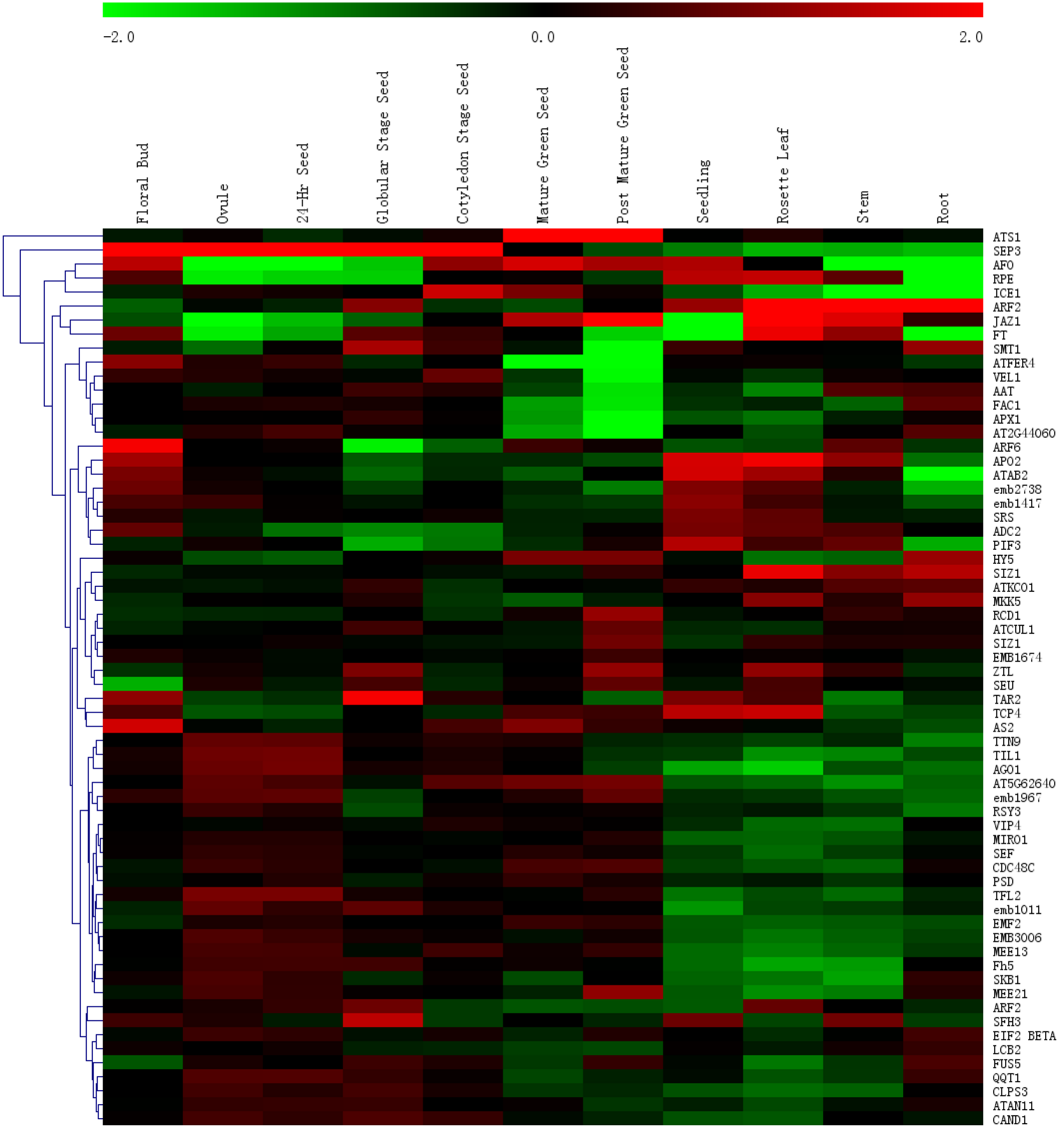
Expression profile of 64 *Arabidopsis* PEDG orthologs during seed development. Seventy *R*. *soongorica*-specific conserved PEDGs and 64 corresponding *Arabidopsis* genes exhibited specific probes on the microarray.

In this study, 287 common conserved, 26 common lost, 70 *R*. *soongorica*-, and 40 sand rice-specific conserved PEDGs were identified (S2 Table). To gain further insight into their expression profiles during seed development, GEO data GSE680 [17] were reanalyzed using GeneSpring software and hierarchical clustering of gene expression signal intensities was conducted to generate a heat map. We opted to focus on the unique phenotypic feature, so only the species-specific conserved and lost genes were illustrated, whereas the expression profiles of the common conserved and lost PEDGs are shown in S2 and S3 Figs. A total of 64 out of 70 *R*. *soongorica*-specific conserved PEDGs were found with specific probes in GSE680. Twenty-eight genes (i.g., *TITAN 9* [*TTN9*], *ARGONAUTE 1* [*AGO1*], and *TERMINAL FLOWER 2* [*TFL2*]) showed high expression in the early stages of seed development, such as in the unfertilized ovules and seeds containing zygotes (24-Hr Seed), and they were down-regulated in the seedling, rosette leaf, and stem (Fig 7). *STEROL METHYLTRANSFERASE 1* (*SMT1*), *FERRITIN 4* (*ATFER4*), *VERNALIZATION INSENSITIVE 3-LIKE 1* (*VEL1*), *ASPARTATE AMINOTRANSFERASE* (*AAT*), *EMBRYONIC FACTOR 1* (*FAC1*), *ASCORBATE PEROXIDASE 1* (*APX1*), and *AT2G44060* showed reduced expression levels, whereas *RADICAL-INDUCED CELL DEATH 1* (*RCD1*), *CULLIN 1* (*ATCUL1*), small ubiquitin-related modifier (SUMO) E3 ligase *SIZ1* exhibited high expression levels in post mature green seed. In particular, *SEPALLATA 3* (*SEP3*) was up-regulated in the floral bud, ovule, zygote, globular stage and cotyledon stage seed. *ARABIDOPSIS THALIANA SEED GENE 1* (*ATS1*) only exhibited high expression levels in mature and post mature green seed.

**Fig 8:**
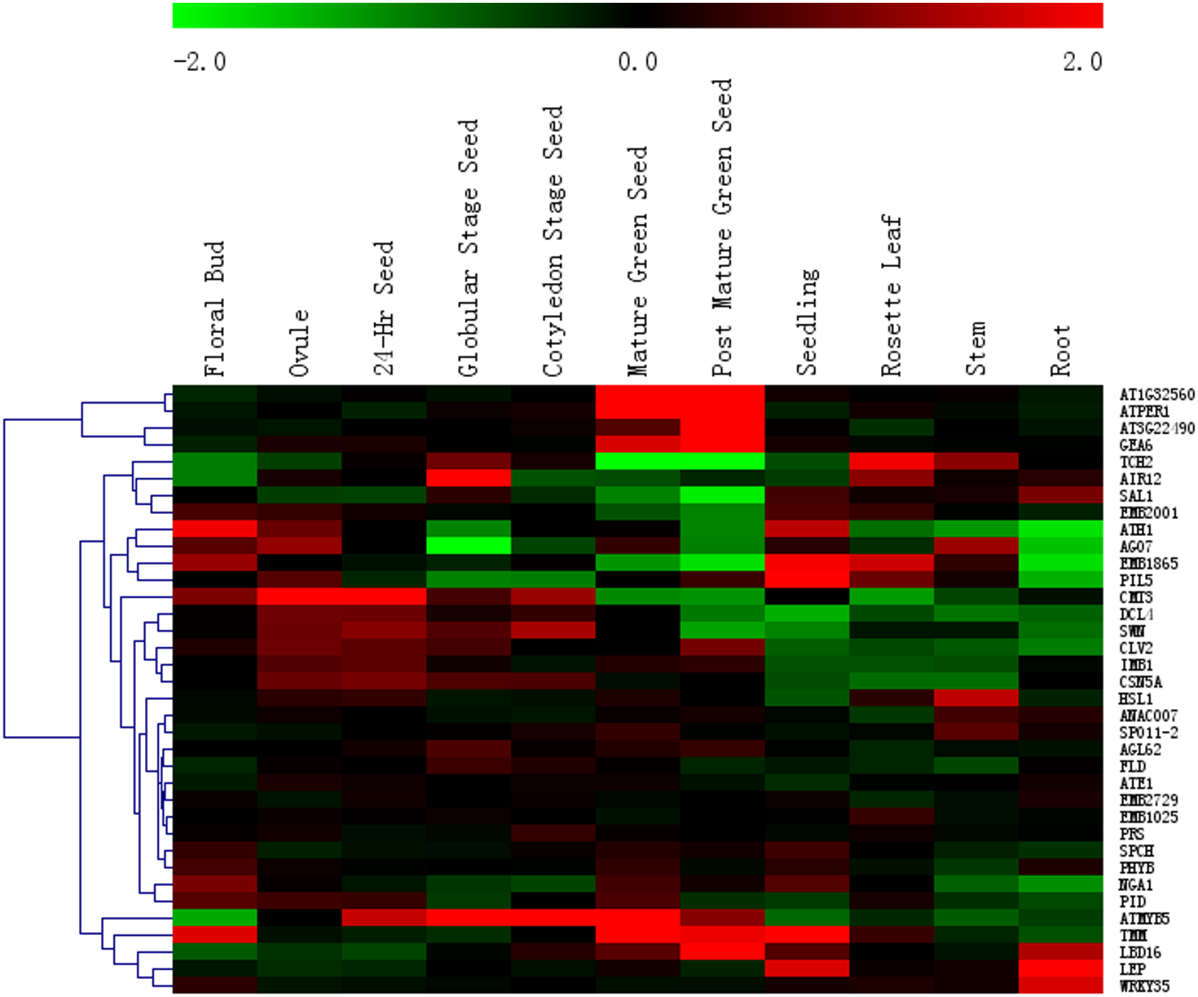
Expression profile of 36 *Arabidopsis* PEDG orthologs during seed development. Forty sand rice-specific conserved PEDGs and 36 corresponding *Arabidopsis* genes showed specific probes on the microarray.

For the 40 sand rice-specific conserved PEDGs, 36 genes with corresponding probes were extracted; the resulting heat map exhibited a diverse expression pattern (Fig 8). Four genes (*AT1G32560*, *1-CYSTEINE PEROXIREDOXIN 1* [*ATPER1*], *AT3G22490*, *EARLY METHIONINE-LABELLED 6* [*EM6*/*GEA6*]) showed high expression levels in mature and post mature green seeds but little signals in other tissues. These findings were consistent with their function as late embryogenesis abundant proteins (LEAs), except for *ATPER1*. *ATMYB5*, which is involved in seed coat formation, mucilage synthesis, and trichome branching [18], showed high expression from the zygote to the post mature green seed. Our unpublished data showed that branch formation was initiated in three-day-old sand rice seedlings. Therefore, the expression profile in three-day-old seedling after imbibition should provide some clues to the genetic base of branch priority. In 36 sand rice PEDGs, *TOO MANY MOUTHS* (*TMM*), *LEAFY PETIOLE* (*LEP*), *EMBRYO DEFECTIVE 1865* (*EMB1865*), *PHYTOCHROME INTERACTING FACTOR 3-LIKE 5* (*PIL5*) exhibited high expression levels, whereas *DICER-LIKE 4* (*DCL4*) exhibited low expression levels in seedlings.

## Discussion

Caryophyllales species comprise approximately 6% diversity of the angiosperm species, and they are known for their extraordinary variety in growth forms and ecological adaptations [19]. *R*. *soongorica* and sand rice are located at different niches and show repeated adaptation to recurring or multiple environmental stresses in the desert ecosystem. Combined with unique phenotypic features, these two species under the order of Caryophyllales are noteworthy and merit the investigation of their gene and genome evolution. The published transcriptomes of *R*. *soongorica* and sand rice paved the way for genome-wide evolution analysis [1,3]. In the present report, gene conservation and loss were dissected by comparisons with the well-annotated *Arabidopsis* genome, and the monocot species *O*. *sativa* was also included to strictly cluster orthologous genes. Moreover, only the sub-cluster that contained the *B*. *vulgaris* ortholog was used to identify the conserved or lost genes because of its phylogenetic relationships with *R*. *soongorica* and sand rice [3]. Notably, the newly evolved genes fall out of the scope of the approach used in this report.

GC content is suggested to play a crucial role in genome function and species ecology [20]. Studies on the genomic composition, especially for non-model plants, which accounts for a large proportion of angiosperm, will enable us to deepen the existing knowledge on the relationship between genomic architecture and organization and adaptations to different ecosystems [20]. An extensive survey of genomic composition across 239 monocot species demonstrated a strong association between variations in GC content and ecological relevance [20]. Higher GC content is significantly associated with ecological distribution and increased tolerance to temperature extremes in monocot species [20]. In our four conserved and lost gene sets, the GC contents of the rice genes were obviously higher than those of the genes from the other species (Figs 3 and 4). This finding was consistent with previous finding that of the Poaceae species, which hold the highest GC contents among land plants [20]. However, a similar association between GC content and climate variations was not found in this study. The GC contents of the *R*. *soongorica* and sand rice genes clustered in our sets were even lower than those of the *Arabidopsis* genes (Figs 3 and 4). This result was due to at least two reasons. First, transcriptome data were used, from which unigenes could be partially assembled. As shown in Figs 3 and 4, the protein lengths of the *R*. *soongorica* and sand rice genes were significantly shorter than those of the other three plants with sequenced genomes. Constructing draft genomes for these two species in the future would resolve this issue precisely. Second, the possibility that a different association exists in dicots cannot be ruled out. A large-scale survey of GC content across a vast majority of dicot species is necessary to test the relationship between genomic composition and ecological adaptations to various environments.

Desiccation tolerance in vegetative tissues of resurrection plants also occurs in pollen grains and orthodox seeds, but not in recalcitrant seeds, which exhibit sensitivity to water loss and only can survive for a short period [21–24]. Large scale transcriptome profiling of dehydration and rehydration tissues of different resurrection plants has shown that many core dehydration-related genes are shared with the orthodox seeds, but their expression patterns are differed from those in the desiccation-sensitive species, namely the recalcitrant seeds [21,25–30]. A recent genomic analysis of a resurrection plant *Boea hygrometrica* did not identify any genome structure specific for desiccation tolerance [30]. This evidence further supports the statement that vegetative desiccation tolerance employs the preexisting genetic elements but alters their expression patterns to provide more effective protection in resurrection plants [21,29,30]. In the present study, 5441 genes were shared among resurrection plant *R*. *soongorica* and other four species (Fig 2). GO annotation revealed that 37 biological process terms were overrepresented (*p* < 0.01; Fig 5). Some GO terms, such as photosynthesis, carbohydrate metabolic process, nitrogen metabolic, lipid metabolic, and response to stress, were also enriched in published transcriptomes of resurrection plants [21,27,29,30]. Eleven GO terms were overlapped with the desiccation tolerance associated transcriptome in *Arabidopsis* seeds [26]. Moreover, we found that 168 *R*. *soongorica* transcripts out of 5441 genes show differential expression patterns in drought-treated transcriptome [4]. These results demonstrated that desiccation tolerance mechanisms similar to those of resurrection plants and seeds are employed by *R*. *soongorica* and sand rice to survival against water shortage in desert ecosystems.

In all four gene sets, the biological process term “post-embryonic development” was overrepresented (*p* < 0.01; Figs 5 and 6). A total of 264 common conserved, 24 common lost, 64 *R*. *soongorica*-, and 36 sand rice-specific conserved PEDGs were found, of which each *Arabidopsis* ortholog has a specific ATH1 probe. A diverse expression pattern was found in all four *Arabidopsis* PEDG sets during seed development (Figs 7 and 8 and S2 Fig). By re-analyzing the cellular localization and molecular function of species-specific PEDGs, we found that the *R*. *soongorica* genes were mostly located in the nucleus (*p* = 3e-06) and cytosol (*p* = 6e-05) and functioned through protein binding (*p* = 3.5e-15). By contrast, the sand rice genes were enriched in the nucleus (*p* = 0.00097) and involved in DNA binding (*p* = 1.2e-06). Out of 64 *R*. *soongorica*-specific conserved PEDGs, three well-known genes showed high expression levels in post mature green seed (Fig 7). RCD1 contains a poly(ADP-ribose) polymerase domain and a WWE protein-protein interaction domain and is involved in abscisic acid (ABA), ethylene, and jasmonate signaling and the regulation of many stress-responsive genes [31–33]. ATCUL1 is an essential component of ubiquitin ligase complex in mediating protein degradation and plays a critical role in diverse cellular processes [34–39]. In *Arabidopsis*, SIZ1 mediated SUMO modification is involved in the regulation of several stress response, such as ABA signaling, phosphate deficiency response, and drought tolerance [40–42]. These results indicated that the core genetic modules implicated in environmental adaptation are conserved during the speciation and evolution of *R*. *soongorica*.

Dehydration-induced accumulation of LEA transcripts and proteins is a shared response in desiccation tolerance and desiccation sensitive plants [21,23,24,43]. Among the 36 sand rice-specific conserved PEDGs (Fig 8), three LEA genes exhibited high expression levels in mature and post mature green seeds. These results lead to the hypothesis that some LEA genes are specifically selected to mediate the acquisition of vegetative dehydration tolerance in sand rice. Five PEDG genes (*TMM*, *LEP*, *EMB1865*, *PIL5*, and *DCL4*) showed evidently up- or down-regulated patterns in the seedling than in the other stages. In *Arabidopsis*, *LEP* mutation results in loss of leaf petiole and aberrant inflorescence branching and silique shape [44,45]. Thus, *LEP* should be considered as a possible candidate gene involved in the regulation of branch priority in sand rice. The detection of its expression profiles during seed development and germination may provide more evidence for its precise function. Meanwhile, *DCL4* is essential for small RNA processing [46], and it is down-regulated in seedlings. Although no direct evidence is available on the involvement of DCL4 in *Arabidopsis* branching, the role of small RNA should be considered when elucidating the genetic base of branch priority.

## Supporting Information

S1 Fig GO annotation of the 5441 conserved genes common to both *R*. *soongorica* and sand rice. (TIF)

S2 Fig Expression profiles of the common conserved *Arabidopsis* PEDG orthologs during seed development. A total of 287 PEDGs were identified to be conserved to both *R*. *soongorica* and sand rice. A total of 264 corresponding *Arabidopsis* genes showed specific probe on the microarray.
(TIF)

S3 Fig Expression profiles of the common lost *Arabidopsis* PEDG orthologs during seed development. A total of 26 PEDGs were identified to be lost to both *R*. *soongorica* and sand rice. A total of 24 corresponding *Arabidopsis* genes showed specific probe on the microarray.
(TIF)

S1 Table Detailed *p* values of the pairwise *t* test. (DOCX)

S2 Table Lists of PEDGs in four different gene sets. (XLSX)
