# Supplementary figures and images for: Different Sets of Post-Embryonic Development Genes Are Conserved or Lost in Two Caryophyllales Species (Reaumuria soongorica and Agriophyllum squarrosum)
Source: PLoS One. 2016 Jan 27;11(1):e0148034. doi: 10.1371/journal.pone.0148034 (PMC4729483; doi:10.1371/journal.pone.0148034)

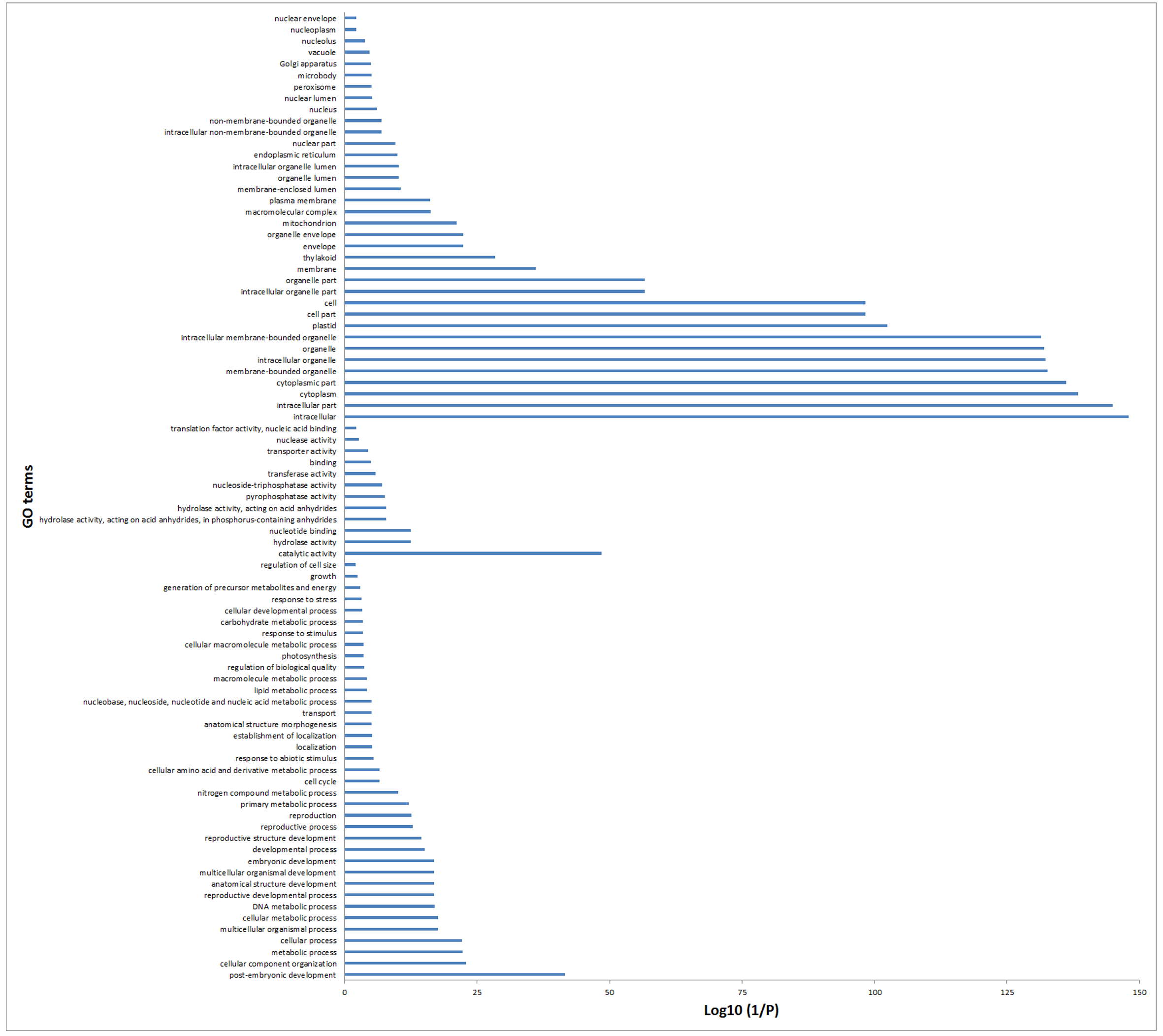

Supplement: S1 Fig — (TIF) [file pone.0148034.s001.tif]

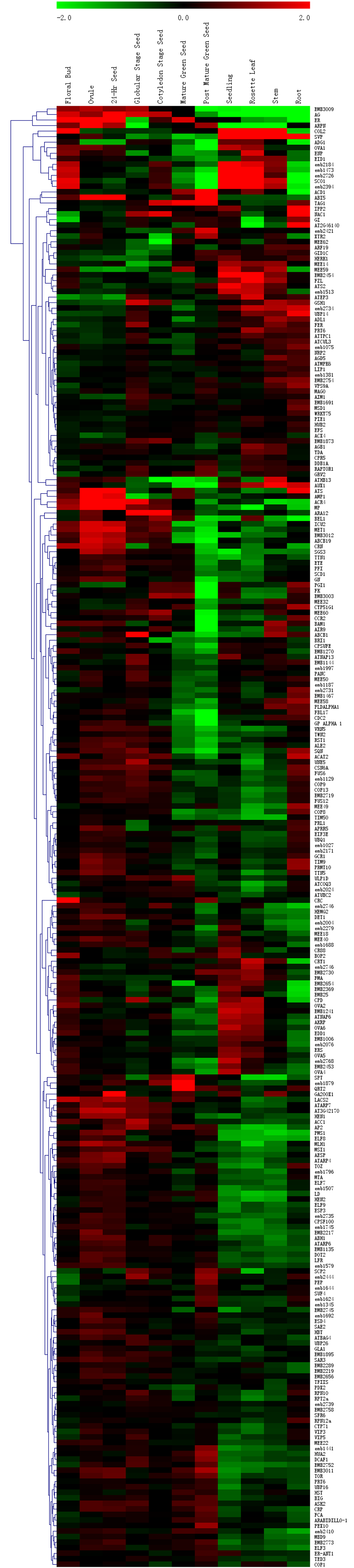

Supplement: S2 Fig — A total of 287 PEDGs were identified to be conserved to both R. soongorica and sand rice. A total of 264 corresponding Arabidopsis genes showed specific probe on the microarray. (TIF) [file pone.0148034.s002.tif]

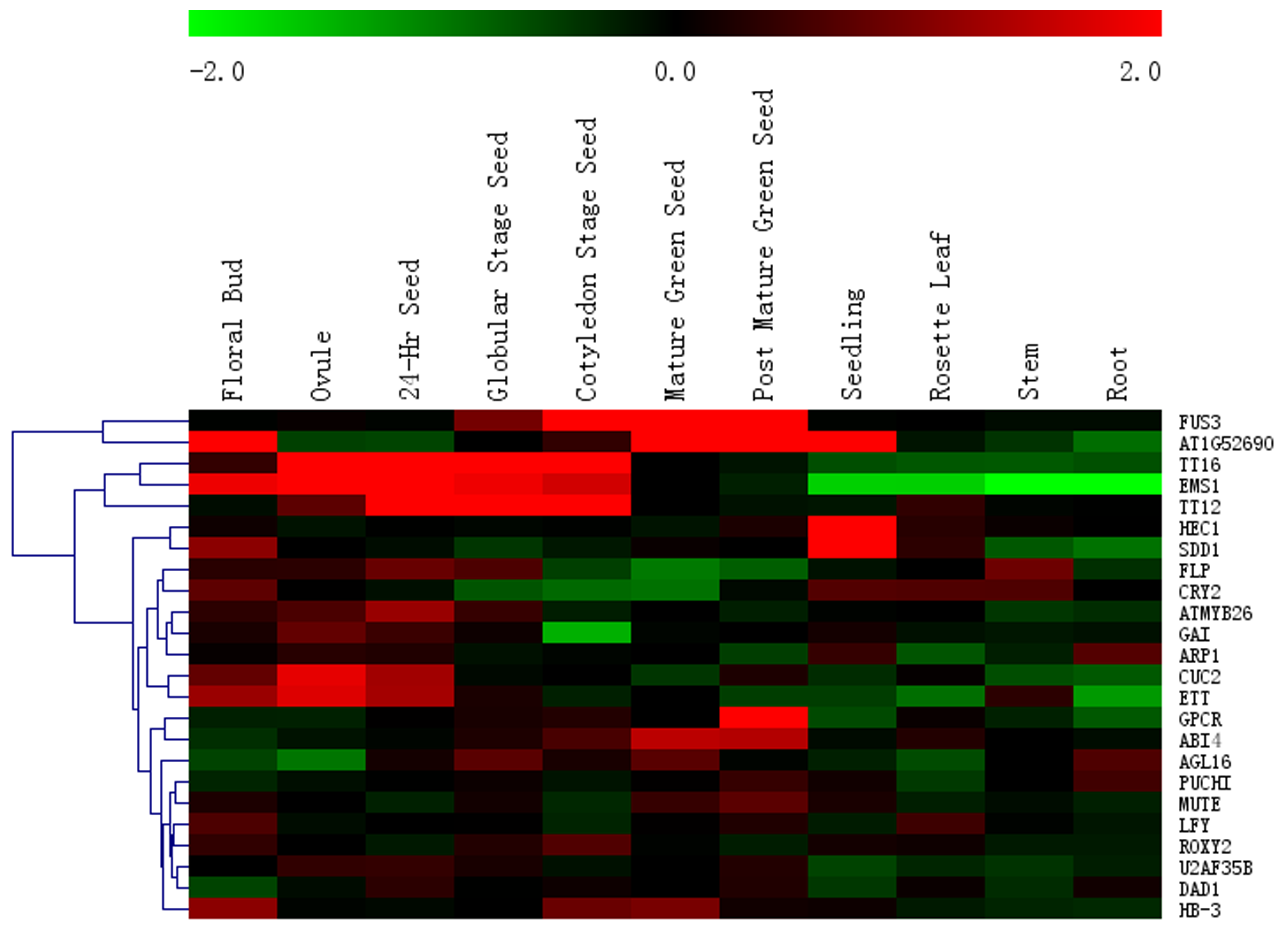

Supplement: S3 Fig — A total of 26 PEDGs were identified to be lost to both R. soongorica and sand rice. A total of 24 corresponding Arabidopsis genes showed specific probe on the microarray. (TIF) [file pone.0148034.s003.tif]
