# Supplementary material for: Different Sets of Post-Embryonic Development Genes Are Conserved or Lost in Two Caryophyllales Species (Reaumuria soongorica and Agriophyllum squarrosum)
Source: PLoS One. 2016 Jan 27;11(1):e0148034. doi: 10.1371/journal.pone.0148034 (PMC4729483; doi:10.1371/journal.pone.0148034)
Supplement: S1 Table — (DOCX) [file pone.0148034.s004.docx]

**Table S1** Detailed *p* values of the pairwise *t* test**.**

5441 genes protein length

|  | At (494.52) | Os (447.54) | Bv (486.35) | Rs (388.14) | As (448.17) |
| --- | --- | --- | --- | --- | --- |
| At |  | 8.72e-15 | 0.24 | **** | 2.44e-12 |
| Os |  |  | 2.26e-10 | **** | 0.91 |
| Bv |  |  |  | **** | 1.05e-08 |
| Rs |  |  |  |  | **** |

5441 genes Identity

|  | Os (73.17) | Bv (76.05) | Rs (76.66) | As (63.45) |
| --- | --- | --- | --- | --- |
| Os |  | **** | **** | **** |
| Bv |  |  | 3.96e-03 | **** |
| Rs |  |  |  | **** |

5441 genes GC content

|  | At (44.88) | Os (54.61) | Bv (43.46) | Rs (44.41) | As (44.19) |
| --- | --- | --- | --- | --- | --- |
| At |  | **** | **** | **** | **** |
| Os |  |  | **** | **** | **** |
| Bv |  |  |  | **** | **** |
| Rs |  |  |  |  | 7.81e-05 |

560 genes protein length

|  | At (471.94) | Os (474.18) | Bv (470.91) |
| --- | --- | --- | --- |
| At |  | 0.89 | 0.95 |
| Os |  |  | 0.84 |

560 genes Identity

|  | Os (57.89) | Bv (60.58) |
| --- | --- | --- |
| Os |  | 2.48e-03 |

560 genes GC content

|  | At (44.74) | Os (57.32) | Bv (43.11) |
| --- | --- | --- | --- |
| At |  | **** | **** |
| Os |  |  | **** |

1912 genes protein length

|  | At (469.65) | Os (420.01) | Bv (457.26) | Rs (380.77) |
| --- | --- | --- | --- | --- |
| At |  | 3.96e-07 | 0.27 | **** |
| Os |  |  | 1.48e-04 | 5.75e-07 |
| Bv |  |  |  | 1.89e-15 |

1912 genes Identity

|  | Os (60.03) | Bv (62.91) | Rs (63.43) |
| --- | --- | --- | --- |
| Os |  | 8.71e-11 | 1.51e-14 |
| Bv |  |  | 0.23 |

1912 genes GC content

|  | At (44.60) | Os (53.08) | Bv (43.60) | Rs (44.50) |
| --- | --- | --- | --- | --- |
| At |  | **** | **** | 0.26 |
| Os |  |  | **** | **** |
| Bv |  |  |  | **** |

723 genes protein length

|  | At (468.97) | Os (486.83) | Bv (493.09) | As (452.94) |
| --- | --- | --- | --- | --- |
| At |  | 0.41 | 0.70 | 2.08e-03 |
| Os |  |  | 0.68 | 2.05e-02 |
| Bv |  |  |  | 8.93e-03 |

723 genes Identity

|  | Os (55.38) | Bv (60.03) | As (59.46) |
| --- | --- | --- | --- |
| Os |  | 1.69e-10 | 1.43e-08 |
| Bv |  |  | 0.41 |

723 genes GC content

|  | At (44.83) | Os (57.99) | Bv (42.94) | As (43.89) |
| --- | --- | --- | --- | --- |
| At |  | **** | **** | 1.81e-07 |
| Os |  |  | **** | **** |
| Bv |  |  |  | 1.88e-07 |
